# Validation of a novel reconstruction method of laparoscopic gastrectomy for proximal early gastric cancer: a systematic review and meta-analysis

**DOI:** 10.1186/s12957-020-01993-7

**Published:** 2020-08-18

**Authors:** Yixin Xu, Jie Gao, Yibo Wang, Yulin Tan, Cheng Xi, Nianyuan Ye, Dapeng Wu, Xuezhong Xu

**Affiliations:** 1 Department of General Surgery, Changzhou Wujin People’s Hospital Affiliated to Jiangsu University, The Wujin Clinical College of Xuzhou Medical University, Changzhou, Jiangsu Province China; 2 Department of General Surgery, Kunshan Traditional Chinese Medicine Hospital, Kunshan, Jiangsu China; 3 Department of Endoscopy, Jiangsu Provincial Hospital of Traditional Chinese Medicine, Nanjing, Jiangsu China

## Abstract

**Background:**

Recently, a novel surgical procedure, named as laparoscopic proximal gastrectomy (LPG) with double-tract reconstruction (DTR), has been reported to provide surgical benefits in the treatment of proximal early gastric cancer (EGC) over traditional laparoscopic total gastrectomy (LTG). These benefits include a lower incidence of some surgical complications and better postoperative nutritional status. However, the number of relevant studies is still too low to validate such benefits. Therefore, this systematic review and meta-analysis aimed to assess the surgical features, complications, and postoperative nutritional status of LPG with DTR in comparison to those of LTG.

**Methods:**

Online databases (PubMed, Web of Science, Cochrane Library, and EMBASE) were scoured for relevant studies published by April 2020. The quality assessment of the included articles was evaluated using the Newcastle-Ottawa scale. Egger’s test was utilized to assess publication bias.

**Results:**

Nine studies (687 patients) were enrolled for this meta-analysis, and we found that LPG with DTR and LTG had similar surgical features. However, LPG with DTR was superior to LTG in the incidence of reflux syndrome [OR = 0.185; 95%CI 0.083, 0.414; *P* = 0.000], postoperative nutritional status (hemoglobin [WMD = − 2.326; 95%CI − 4.491, − 0.160; *P* = 0.035], vitamin B12 [WMD = − 13.072; 95%CI − 22.850, − 3.294; *P* = 0.009], and body weight [WMD = − 3.514; 95%CI − 5.579, − 1.449; *P* = 0.001]).

**Conclusions:**

LPG with DTR has better performance in the incidence of reflux syndrome and postoperative nutritional status compared with LTG. This surgical procedure may therefore have more benefits for patients with proximal EGC.

## Introduction

In 2018 alone, gastric cancer (GC) had approximately 1 million new cases and 800,000 deaths, implying 1 in 10 cancer cases and deaths [1]. Meanwhile, the incidence of proximal early gastric cancer (EGC) in both developed and developing countries has continued to rise [2, 3]. The optimum therapeutic method for proximal EGC is surgery. With the current advances in medical technology, almost all abdominal surgery can be performed using laparoscopy.

The laparoscopic surgical procedures for proximal EGC usually include laparoscopic proximal gastrectomy (LPG) and laparoscopic total gastrectomy (LTG).

The reconstruction method of conventional LPG is esophagogastrostomy, which is often associated with serious postoperative complications, including anastomotic stenosis and reflux syndrome [4]. Therefore, LTG with Roux-en-Y esophagojejunostomy is the preferred surgical procedure for proximal EGC [5].

**Fig. 1:**
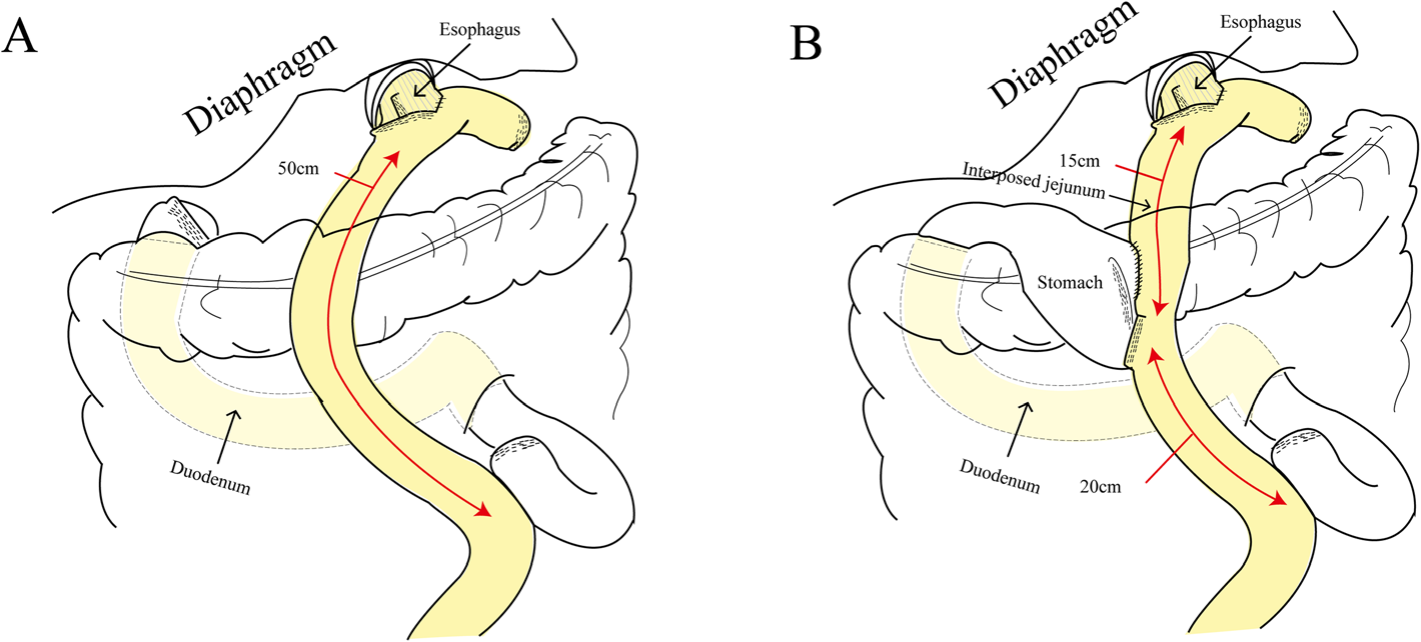
Schema of reconstruction. **a** Laparoscopic total gastrectomy with Roux-en-Y esophagojejunostomy. **b** laparoscopic proximal gastrectomy with double-tract reconstruction

The reconstruction method of LTG is shown in Fig. 1a [6]. However, LTG still has several disadvantages, including food intake restriction and long-term malnutrition (anemia caused by vitamin B12 deficiency and low body weight). Due to these shortcomings, patients who have undergone LTG may suffer low quality of life (QOL), especially for those with relatively long survival time.

A novel reconstruction method of LPG, termed double-tract reconstruction (DTR), has recently emerged. Figure 1b details the DTR procedure which has three anastomoses, including esophagojejunostomy (E-J stomy), jejunogastrostomy (J-G stomy), and jejunojejunostomy (J-J stomy). The procedure of E-J stomy is similar to that of LTG. The distances of E-J stomy to J-G stomy and J-G-stomy to J-J stomy are usually 15 cm and 20 cm, respectively. Theoretically, the remnant stomach should be able to overcome problems such as food intake restriction, and deficiency of vitamin B12 and iron. Meanwhile, the interposed jejunum between E-Jstomy and J-Gstomy can alleviate the reflux syndrome problem.

Since its adoption, LPG with DTR has been constantly compared with LTG in postoperative complications and clinical outcomes. Whether LPG with DTR or LTG offers a better therapeutic value for patients with proximal EGC remains controversial until now. Therefore, we sought to assess the surgical features and clinical outcomes between LPG with DTR and LTG.

## Methods

### Literature search strategy

A systematic literature search for studies comparing the surgical features and clinical outcomes between LPG with DTR and LTG was conducted online. Keywords, such as (laparoscopic) and (proximal) and (“double tract” or “double-tract” or “two tract” or “two-tract”) and (total) and (gastrectomy), were used to search databases including PubMed, Web of Science, Cochrane Library, and EMBASE for relevant articles published by April 30, 2020.

The full articles were carefully reviewed and we subsequently screened bibliographies of the retrieved articles to identify any potential source of relevant studies.

### Study selection

The inclusion criteria included (1) studies included proximal EGC (clinical stage I); (2) patients underwent either LPG with DTR or LTG; (3) patients were categorized into two groups (LPG with DTR or LTG); and (4) surgical features and/or clinical outcomes (postoperative complications and/or nutritional status) were presented. The exclusion criteria included (1) the types of articles were reviews, letters, comments, and case reports; (2) precise data were unavailable in the articles; and (3) animal studies and non-English publications.

### Data extraction and quality assessment

Two independent researchers (Wang and Tan) extracted the data. The information from enrolled studies included the author information, sample size, surgical features, and elements of clinical outcomes. Moreover, in case of any disagreement between the two reviewers, a third investigator (Xi) would adjudicate.

All continuous data would be converted into mean with standard deviation [7] and analyzed using weighted mean differences (WMD) with 95% confidence intervals (CIs) intervals. However, the dichotomous data were measured using odds ratios (ORs).

The quality of the included studies (cohort or case-control) was assessed using the Newcastle-Ottawa scale (NOS). The NOS evaluates studies using a scale from 0 to 9, and studies that scored > 6 were considered high quality [8].

### Outcomes of interest

The surgical features were initially evaluated between two groups, before assessing the postoperative complications and nutritional status (1 year postoperatively).

### Statistical analysis

Heterogeneity between studies was tested using Cochran *Q*, whereas Higgins’ *I*^2^ statistics were used to assess heterogeneity among studies [9]. If the value of *I*^2^ was more than 50%, and the *P* < 0.05, indicating the existence of significant heterogeneity, then a random-effect model was selected. Otherwise, a fix-effect model would be chosen. If the heterogeneity of included studies was considered significant (higher than 50%), a sensitivity analysis would be carried out to identify the source of heterogeneity. Additionally, the Egger’s test was used to assess the publication bias. Statistical significance was set at *P* < 0.05. STATA version 14.0 was used to perform all the meta-analyses.

## Results

### Search strategy

**Fig. 2:**
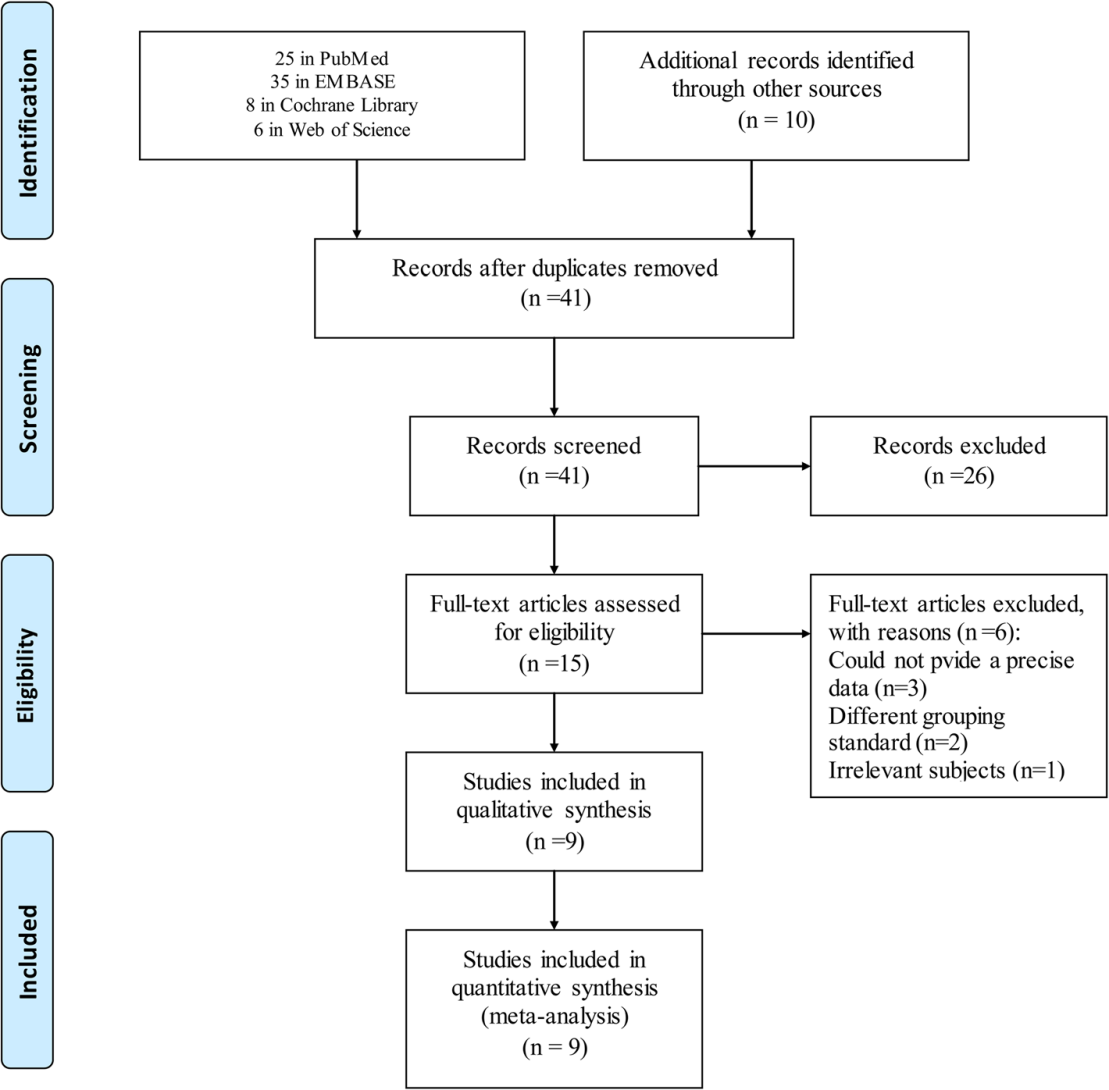
Flow diagram for study selection

Sixty-nine articles were identified including 20 in PubMed, 6 in Web of Science, 8 in Cochrane Library, and 35 in EMBASE. Forty-one articles were identified as duplicates and were removed after screening. Twenty-six articles were excluded, including non-English journals, case reports, or analyses. Those with imprecise data, varying grouping standards, and irrelevant subjects were similarly omitted. Eventually, nine articles were included in the present meta-analysis (Fig. 2).

### Cohort characteristics and quality of the studies

Nine studies eventually enrolled in the present meta-analysis [6, 10–17] are from Asian countries, i.e., 5 in Korea, 3 in Japan, and 1 in China. All studies provided surgical features and complications, while 6 provided postoperative nutritional status. One article got a NOS score of 6, 5 received a NOS score of 7, and 3 scored 8, respectively. Two studies were prospective cohort studies, and others were retrospective cohort studies. All characteristics of the included studies are shown in Table 1.

**Table 1:** Characteristics of studies included in meta-analysis

Author	Year	Country	Sample size (LPG/LTG)	Study type	Surgical features reported	Complications reported	Nutritional status reported	NOS score
Park	2018	Korea	34/46	Retrospective	√	√	√	8
Aburatani	2017	Japan	19/22	Retrospective	√	√	√	7
Nomura	2018	Japan	15/30	Prospective	√	√	n/a	6
Cho	2018	Korea	38/42	Retrospective	√	√	√	7
Ahn	2013	Korea	43/50	Retrospective	√	√	n/a	7
Sugiyama	2018	Japan	10/20	Retrospective	√	√	√	8
Jung	2017	Korea	92/156	Retrospective	√	√	√	7
Kim	2016	Korea	17/17	Prospective	√	√	√	8
Wang	2020	China	12/24	Retrospective	√	√	n/a	7

*LPG* laparoscopic proximal gastrectomy with double-tract reconstruction, *LTG* laparoscopic total gastrectomy, *NOS* Newcastle-Ottawa scale

### Surgical features

**Fig. 3:**
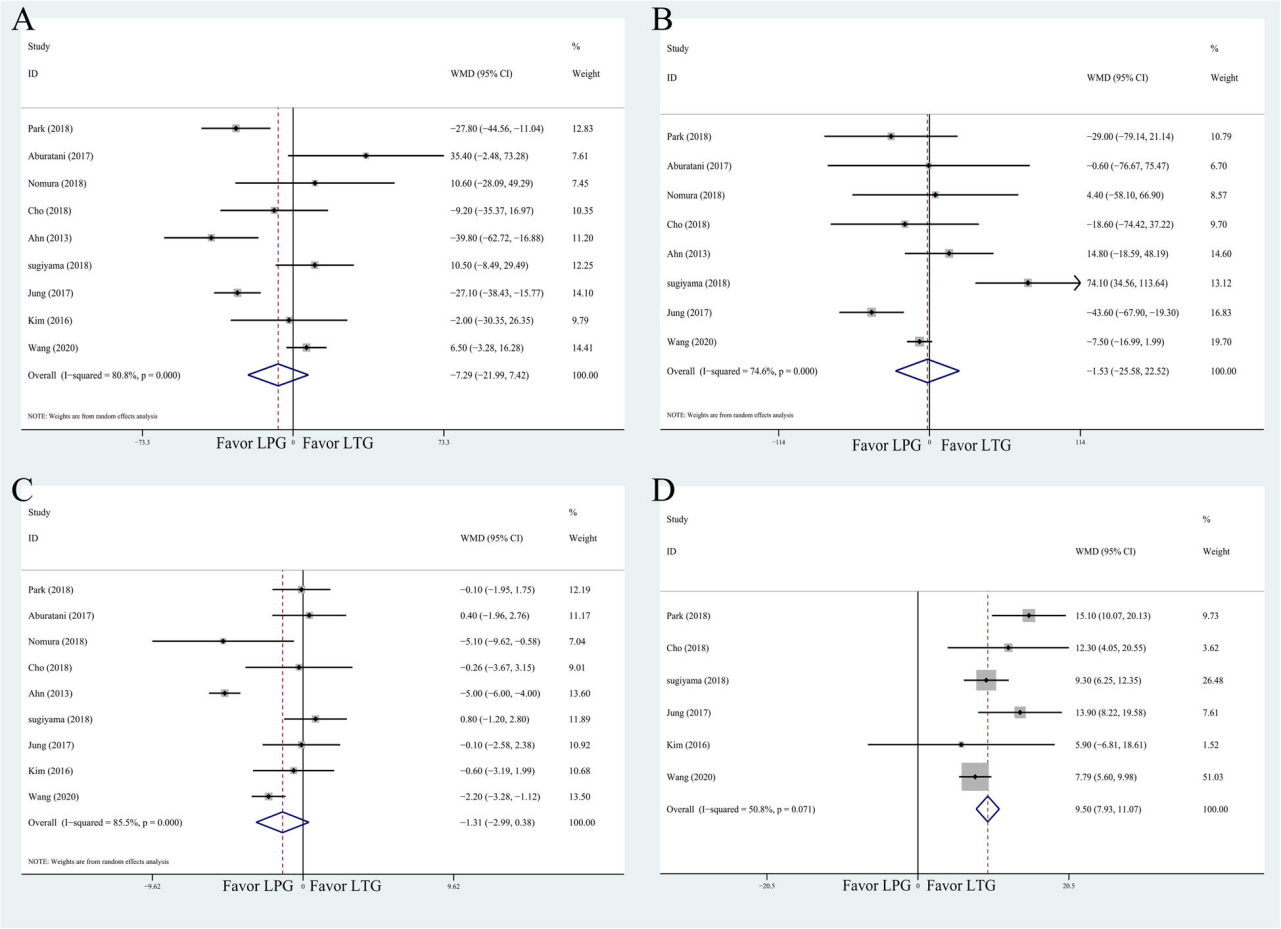
Meta-analysis forest plots for comparison of surgical features. **a** Operation time. **b** Intraoperative blood loss volume. **c** Postoperative hospital stay. **d** Harvested lymph nodes

All data on surgical features are shown in Table 2 and Fig. 3.

**Table 2:** Meta-analysis results of surgical features

Measured outcome	Studies	Patients	WMD	95%CI		*P*	Heterogeneity test		*P* > |*t*|
							*I*^*2*^	*P*	
Operation time	9	687	− 7.287	− 21.990	7.415	0.331	80.8	0.000	0.796
Intraoperative blood loss	8	653	− 1.531	− 25.580	22.517	0.901	74.6	0.000	0.683
PO hospital stay	9	687	− 1.307	− 2.992	0.378	0.128	85.5	0.000	0.101
Harvested lymph nodes	6	508	9.501	7.933	11.069	0.000	50.8	0.071	0.249

*PO* post operation

#### Operation time

Nine included studies (687 patients) had precise data about the time of operation. The random-effects model was used, due to the significant heterogeneity (*I*^2^ = 80.8%, *P* = 0.000). There was however no difference between two groups (WMD = − 7.287; 95%CI − 21.990, 7.415; *P* = 0.331) (Table 2 and Fig. 3a).

#### Intraoperative blood loss volume

Eight studies provided data about intraoperative blood loss volume. The difference between two groups was not statistically significant (WMD = − 1.531; 95% CI − 25.580, 22.517; *P* = 0.901). Additionally, the heterogeneity was significant (I^2^=74.6%, *P* = 0.000) (Table 2 and Fig. 3b).

#### Postoperative hospital stay

Due to the significant heterogeneity (*I*^2^ = 85.5%, *P* = 0.000), the random-effects model was used. The result revealed no significant differences between two groups (WMD = − 1.307; 95% CI − 2.992, 0.378; *P* = 0.128) (Table 2 and Fig. 3c).

#### Harvested lymph nodes

The fixed-effect model was used due to moderate heterogeneity exhibited (*I*^2^ = 50.8%, *P* = 0.249). The result was in favor of the LTG group (WMD = 9.501; 95% CI 7.933, 11.069; *P* = 0.000) as shown in Table 2 and Fig. 3d.

### Postoperative complications

**Fig. 4:**
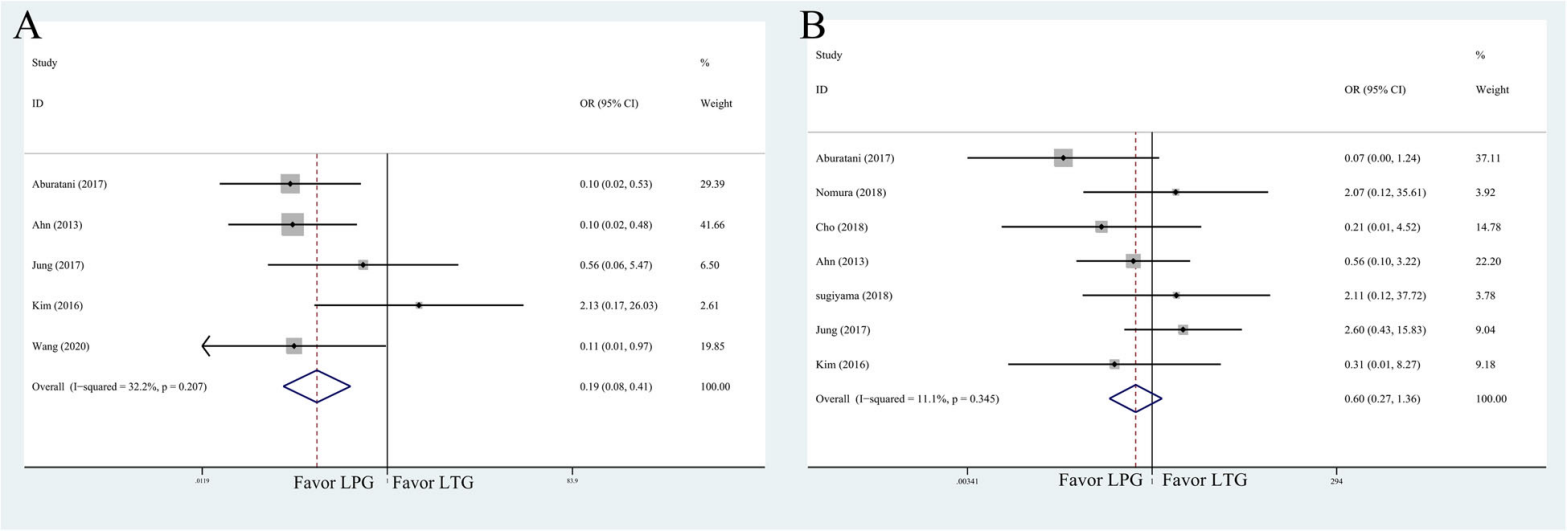
Meta-analysis forest plots for comparison of postoperative complications. **a** Reflux syndrome. **b** Anastomotic stenosis

The results of postoperative complications are presented in Table 3. Additionally, the forest plots for comparison of reflux syndrome and anastomotic stenosis are shown in Fig. 4a, b.

**Table 3:** Meta-analysis results of postoperative complications

Measured outcome	Studies	Patients	OR	95% CI		*P*	Heterogeneity test		*P* > |*t*|
							*I*^*2*^	*P*	
Overall complications	7	601	0.634	0.334	1.201	0.162	55.2	0.037	0.636
Anastomotic leakage	6	480	0.783	0.298	2.060	0.620	0.0	0.764	0.352
Pancreatic fistula	5	450	0.562	0.211	1.499	0.250	13.3	0.326	0.341
Reflux syndrome	5	450	0.185	0.083	0.414	0.000	32.2	0.207	0.169
Anastomotic stenosis	7	571	0.604	0.269	1.357	0.222	11.1	0.345	0.513

#### Overall complications

Seven studies (601 patients) provided data with significant heterogeneity of overall complications (*I*^2^ = 55.2%, *P* = 0.037). However, the incidences of overall surgical complications between two groups were not statistically different (OR = 0.634; 95% CI 0.334, 1.201; *P* = 0.162) (Table 3).

#### Analysis of complications by subgroup

In the subgroup analysis, the differences in the incidences of anastomotic leakage (OR = 0.783; 95% CI 0.298, 2.060; *P* = 0.620), pancreatic fistula (OR = 0.562; 95% CI 0.211, 1.499; *P* = 0.250), and anastomotic stenosis (OR = 0.604; 95% CI 0.269, 1.357; *P* = 0.222) were not statistically significant.

Conversely, the incidence of reflux syndrome was significantly higher in the LTG group (OR = 0.185; 95% CI 0.083, 0.4146; *P* = 0.000). Based on the moderate heterogeneity (*I*^2^ = 32.2%, *P* = 0.207), the fixed-effects model was used.

### Postoperative nutritional status

To assess the postoperative nutritional status between LPG with DTR and LTG, the decreased rate of seven variables measured preoperatively and 1 year after surgery was selected. These variables included albumin, hemoglobin, total protein, vitamin B12, total cholesterol, iron, and body weight loss.

**Fig. 5:**
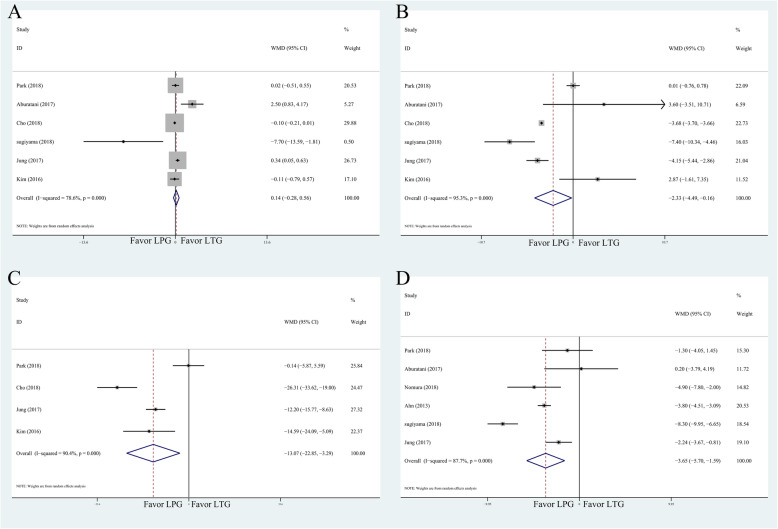
Meta-analysis forest plots for comparison of postoperative nutritional status. **a** Albumin. **b** Hemoglobin. **c** Vitamin B12. **d** body weight

All results of postoperative nutritional status are shown in Table 4. Moreover, the overall effects size of albumin, hemoglobin, vitamin B12, and body weight were shown in Fig. 5.

**Table 4:** Meta-analysis results of postoperative nutritional status

Measured outcome	Studies	Patients	OR	95% CI		*P*	Heterogeneity test		*P* > |*t*|
							*I*^*2*^	*P*	
Albumin	6	513	0.139	− 0.282	0.560	0.517	78.6	0.000	0.688
Hemoglobin	6	513	− 2.326	− 4.491	− 0.160	0.035	95.3	0.000	0.344
Total protein	6	513	0.462	− 0.664	1.588	0.422	55.1	0.000	0.209
Vitamin B12	4	442	− 13.072	− 22.850	− 3.294	0.009	90.4	0.000	0.771
Cholesterol	4	449	− 0.610	− 5.863	4.644	0.820	49.0	0.000	0.927
Iron	3	194	3.000	− 25.634	31.634	0.837	56.8	0.000	0.958
Body weight	6	537	− 3.514	− 5.579	− 1.449	0.001	89.8	0.000	0.870

The overall effects of albumin level, total protein, total cholesterol, and iron did not favor either LPG or LTG groups. However, it showed that hemoglobin, vitamin B12, and body weight were significantly lower in LTG group (Table 4).

### Publication bias and sensitivity analysis

The publication bias was assessed in every element using Egger’s test, but no publication bias was revealed. Similarly, no studies with potential heterogeneity were revealed from the Galbraith plot, used to measure heterogeneity.

## Discussion

With the advancements of endoscopic technology, several physicians prefer to treat patients with proximal EGC. The endoscopic therapeutic procedures for proximal EGC mainly include endoscopic mucosal resection (EMR) and endoscopic submucosal dissection (ESD) [18, 19]. However, both of them have their respective limitations. For example, EMR is only recommended for the treatment of ulcer-free EGC, which should be less than 20 mm in diameter [20]. However, the incidence of lymph node metastasis of lesions limited to mucosa varies from 3 to 5%. Meanwhile, the incidence increases to 16–25% for those lesions deep into submucosa [21–23]. Due to the relatively high risk of lymph metastasis, the optimal therapeutic method for proximal EGC is still curative gastrectomy, which includes LPG and LTG [24–26]. Although the conventional LPG is considered more function-preserved, surgeons prefer LTG over LPG due to the severe postoperative complications (anastomotic stenosis and reflux syndrome) caused by the reconstruction method of esophagogastrostomy [27, 28]. The common surgical procedure for proximal EGC patients is therefore LTG. However, LTG is far from satisfactory. Besides, patients who have undergone LTG often exhibit several symptoms such as low body weight, anemia, food intake restriction, diarrhea, and dumping [29, 30]. Recently, surgeons have been gradually accepting LPG with DTR as it is thought to overcome the shortcomings of conventional LPG and LTG [14, 31]. However, there are still controversies about which surgical procedure is the best choice for proximal EGC patients.

There are three elements of optimal surgical procedure that should be considered before deciding. These include oncological safety, anastomosis-related complications, and functional benefits.

First, oncological safety means the lymphadenectomy technique, the margin of resection and the quantity of the lymph nodes (LNs) retrieved. The clinical diagnosis of EGC is not quite accurate due to some technical constraints. Occasionally, lymph nodes metastasis occurs in patients diagnosed with EGC. Lee et al. reported that of 1308 patients with clinical EGC, 93 patients had group 1 and 33 had group 2 lymph node metastasis. Extended lymphadenectomy is recommended for EGC patients with tumor located in the proximal third of the stomach [32]. However, patients who underwent LPG with DTR always received D1+ lymphadenectomy. The data provided by six articles enrolled in the present study on the quantity of retrieved LNs, favored the LTG group. Hence, the probability metastatic lymph nodes being missed in patients undergoing LPG with DTR is relatively higher. To compensate for this disadvantage, precise clinical diagnosis or more radical procedure may be needed to prevent missed diagnosis of patients with lymph node metastasis.

Secondly, the aim of performing LPG for proximal EGC patients is to preserve the gastric reservoir with its secretory function [33]. The conventional reconstruction method of LPG is direct esophagogastrostomy, which is simple and requires only one anastomosis. However, this kind of anastomosis means the loss of the lower esophageal sphincter and the acute angle of the lesser curvature. Moreover, it may cause severe anastomosis-related complications, reflux syndrome, and anastomotic stenosis [34]. There are several anti-reflux and anti-stenosis procedures, including fundoplication, gastric tube formation, pyloroplasty, and esophagopexy with a crural repair. However, these methods are unfavorable because they all employ esophagogastrostomy; the primary cause of reflux and stenosis [35, 36].

Fortunately, there is a better alternative reconstruction method, Roux-en-Y type E-Jstomy. This effective method of reconstruction involves performing either DTR or jejunal interposition, following LPG. Although jejunal interposition is widely accepted by surgeons in the open surgery, its standard procedure includes three anastomoses and the formation of a pedicled jejunal flap. It is therefore considered to be technically complex and has not yet been accepted in laparoscopic surgery [37].

Secondly, in the procedure of DTR, the distance from esophagojejunostomy (E-Jstomy) to gastrojejunostomy (G-Jstomy) varies with the habits of the surgeon. For example, Ahn had reported 10 cm and Nomura reported 15 cm [11, 14]. Jung et al. found that the incidence of reflux syndrome was rather high when the length of the interposed jejunum was 10 cm [12]. Theoretically, the incidence of reflux syndrome decreases as the length of interposed jejunum increases. But it has already been proven that the length of over 20 cm is not associated with a lower rate of reflux syndrome [38, 39]. As a result, the optimal distance from E-Jstomy to G-Jstomy could be 15 cm.

Our findings revealed that the surgical features of LPG with DTR were not statistically different from those of LTG, indicating that the technical complexities between both procedures are comparable. Moreover, the incidence of reflux syndrome was significantly lower in LPG with DTR group. This result clearly identified the superiority of DTR in anti-reflux function.

Finally, functional benefits are concentrated mainly on the absorption of vitamin B12 and serum iron, and body weight maintenance. Previous studies have revealed that LPG with DTR performed better than LTG in nutrient absorption function [10, 11, 15]. The main principle is that serum iron absorption is related to the processes of food bypass in the duodenum and proximal jejunum [40]. LPG with DTR maintains the food pathways which could potentially influence the iron metabolism. In the present study, we found that LPG with DTR had superiority of maintenance of hemoglobin, vitamin B12, and body weight.

However, Cho et al. found LPG with DTR, and LTG to have similar long-term nutritional outcomes [6]. Ahn et al. showed that in the reconstructed digestive tract of patients who underwent LPG with DTR, only 60% of food intake went through the remnant stomach, while the other passed directly into the jejunum [11]. Such a quantity of food intake through remnant stomach into duodenum is not sufficient to prevent iron deficiency. In addition, the less oral intake caused by the smaller volume of the remnant stomach after gastrectomy may also affect iron deficiency. Iron deficiency was also observed following distal subtotal gastrectomy, which fully preserves the digestive tract from remnant stomach to duodenum [41]. Therefore, the preservation of distal stomach may be insufficient to prevent iron deficiency.

Vitamin B12 metabolism is significantly affected by the secretion of intrinsic factor released from gastric parietal cells. Besides, gastric parietal cells are basically located in the body of the stomach, much of which may be removed after LPG. Moreover, bile reflux or other cellular pathological changes can also affect the remaining parietal cells [42]. The deficiency of vitamin B12 after the procedure of LPG with DTR may be inevitable. Therefore, further work should determine the optimal surgical procedure is the optimal choice in preserving nutritional function. In the LPG with DTR, the development of remnant gastric cancer is a serious concern. Besides, the incidence of the remnant gastric cancer after LPG is reported to range between 5 and 5.4% [43], compared to 0.4–2.9% after distal gastrectomy [44]. This could be attributed to the relatively high incidence of cancer development in distal stomach. Therefore, the routine endoscopic examination is strongly recommended for patients who have undergone LPG with DTR. Although it is technically difficult to examine the remnant distal stomach, a previous article reported three intubation failures occurred among 43 patients underwent LPG with DTR [11]. However, it is possible for endoscopists to examine the remaining distal stomach after the surgeons have explained the specific DTR procedure. Thus, the regular and repeated communication between endoscopists and surgeons is critical for endoscopic surveillance for patients after undergoing LPG with DTR.

This study had the following limitations. First, patients enrolled in the present study were of different nationalities and had different physical histories. This would result in a significant selection bias. Secondly, the sample size of the included studies is relatively small, which could make our findings unreliable. Finally, although we searched the databases very carefully, articles from western countries were not found. Selection bias may, therefore, exist in our findings as all included studies were from Asia.

## Conclusion

The surgical features in both LPG with DTR and LTG groups are comparable, which translates to similar technical complexity. Although some advantages still need to be confirmed, LPG with DTR is superior to LTG with reference to reflux syndrome incidence and preservation of nutritional function (hemoglobin, vitamin B12, and body weight). For patients with proximal EGC, LPG with DTR may be used as a valuable surgical procedure.

## Data Availability

All the data comes from databases. The author has sorted out all the data and attached to the attachment at the end of the articles.

## Abbreviations

CI: Confidence interval
DTR: Double-tract reconstruction
ESD: Endoscopic submucosal dissection
EGC: Early gastric cancer
EMR: Endoscopic mucosal resection
E-Jstomy: Esophagojejunostomy
GC: Gastric cancer
G-Jstomy: Gastrojejunostomy
HR: Hazard ratio
J-Jstomy: Jejunojejunostomy
LN: Lymph node
LPG: Laparoscopic proximal gastrectomy
LTG: Laparoscopic total gastrectomy
OR: Odds ratio
NOS: Newcastle-Ottawa scale
QOL: Quality of life
WMD: Weighted mean differences
